# Geometry as a Selector of Optical States in Curved Photonic Architectures

**DOI:** 10.3390/polym18182288

**Published:** 2026-09-19

**Authors:** Ion Sandu, Claudiu Teodor Fleaca, Iulia Antohe, Florian Dumitrache, Iuliana Urzica, Iustina Popescu, Marius Dumitru

**Affiliations:** 1 Lasers Department, National Institute for Lasers, Plasma and Radiation Physics, 409, Atomistilor Street, Magurele, 077125 Bucharest, Romania; ion.sandu@inflpr.ro (I.S.); iulia.antohe@inflpr.ro (I.A.); florian.dumitrache@inflpr.ro (F.D.); iuliana.urzica@inflpr.ro (I.U.); 2 Academy of Romanian Scientists (AOSR), Ilfov Street 3, 050044 Bucharest, Romania; claudiu.fleaca@inflpr.ro; 3 Geological Institute of Romania, 1 Caransebes Street, 012271 Bucharest, Romania

**Keywords:** colloidal crystals, self-assembly, photonic heterostructures, Bragg diffraction, light–matter interaction, spectral redistribution

## Abstract

Optical response is commonly regarded as an intrinsic property of a photonic structure. Here we show that, in curved photonic systems, geometry plays a dual role: the photonic architecture defines the optical-state landscape, whereas the illumination–observation geometry determines which optical states become experimentally accessible. Self-assembled, millimetre-scale, free-standing curved silica opals coupled to spherical water droplets on Teflon or planar mirrors reveal simultaneous access to multiple Bragg diffraction bands, together with distance-controlled spectral redistribution, spatially organized halos, and pseudo-collimated beams under different illumination–observation geometries. Natural illumination further reveals optical responses relevant to biological and biomimetic photonic structures. Key aspects of this optical functionality are transferred to a graded curved photonic heterostructure formed by partial polystyrene infiltration of a curved silica opal, demonstrating that the underlying geometrical principle extends beyond liquid-confined systems. Curved photonic architectures therefore provide an experimental platform for geometry-controlled optical-state accessibility.

## 1. Introduction

Modern photonics has been built upon a conceptual separation between geometrical optics and wave optics, as well as between the optical object and its surrounding environment [1]. Conventionally, geometrical optics describes macroscopic light propagation [2], whereas wave optics focuses on periodicity, interference, and Bragg scattering in photonic lattices [3,4,5,6]. Concurrently, the external environment is typically treated as optically secondary—ideally reduced to a weak perturbation to isolate the system’s intrinsic optical response [1]. Although this reductionist paradigm has enabled major advances in optical communication, sensing, metasurfaces, dielectric resonators, and integrated photonics [7,8,9,10], it has also shifted attention away from how geometry itself can actively shape experimentally accessible optical responses.

This separation stems partly from methodological constraints. Planar architectures naturally simplify both experiments and theoretical modelling. A single global surface normal and well-defined incidence conditions make spectroscopic interrogation, angular analysis, and numerical simulations straightforward. Consequently, much of modern photonics—and colloidal photonics in particular—has developed around planar systems, where the optical response is commonly described through the position, width, and intensity of a few Bragg reflections, most often the (111) band [11,12]. In curved photonic architectures, however, the continuous distribution of local surface orientations fundamentally alters the angular phase space of light propagation, enabling optical regimes inaccessible to planar structures [13]. Yet, the longstanding separation of geometrical and wave optics, combined with the experimental isolation of the optical object from its environment, has left the optical consequences of their coupling largely unexplored.

More fundamentally, natural photonic systems rarely operate under planar or environmentally isolated conditions. Instead, biological photonic architectures develop within curved, adaptive environments where geometry, morphology, and optical functionality co-evolve. Butterfly wings, iridescent tissues, biominerals, natural opals, cellular membranes, and bio-colloidal assemblies do not isolate the optical structure from its surroundings. Rather, their optical response emerges from the coupled interaction between geometry, wave propagation, and environmental constraints, producing a remarkable diversity of optical phenomena that continues to inspire photonic design [14,15,16].

Existing synthetic approaches—including colloidal superballs, spherical, cylindrical, and toroidal assemblies, photonic films on curved substrates, mechanically deformed elastic sheets, and additively manufactured structures—successfully reproduce individual aspects of curvature. However, none simultaneously provide the structural quality and geometrical freedom required to experimentally investigate the broader optical consequences of curvature [17,18].

Alternative conceptual strategies—such as transformation optics, gradient-index metamaterials, wavefront-engineered metasurfaces, and dielectric parameter mapping—reproduce selected consequences of curvature through spatial modulation of the refractive index or boundary conditions [9,19,20]. Although highly effective for controlling specific propagation pathways, these approaches remain fundamentally different from genuinely curved photonic architectures, where geometry, periodicity, interfaces, confinement, and the surrounding environment are intrinsically coupled within the same optical object.

In parallel, deterministic fabrication strategies—including electron-beam lithography, UV lithography, and advanced nanofabrication—have largely prioritized structural perfection, reproducibility, and planar optical interrogation [5,9,21,22,23,24]. While this paradigm has enabled extraordinary technological advances, it has also limited the systematic exploration of geometry itself as an active degree of freedom capable of generating new optical functionality.

When these geometrical and environmental constraints are relaxed, curved photonic systems begin to reveal optical responses inaccessible under planar interrogation. In our recent work [13], we reported several unusual phenomena—including blue-beam formation, ghost imaging, and anomalous UV–Vis spectra—that suggested curved photonic architectures support optical regimes fundamentally different from their planar counterparts. Rather than representing isolated optical effects, these observations pointed toward a broader geometrical principle: optical response depends not only on the intrinsic photonic architecture, but also on geometry acting as an active selector of experimentally accessible optical states. This emerging picture, first captured through individual anomalies in Ref. [13], further suggests that the surrounding geometrical and dielectric environment participates directly in determining how these optical states are accessed and redistributed.

Here, we extend this exploration to coupled curved photonic systems formed by millimetre-scale, free-standing opal photonic crystals confined within water droplets. In this framework, optical functionality emerges from the coupled interplay between photonic order, curvature, cavity geometry, interfaces, and the surrounding optical environment. Within this architecture, we distinguish two complementary geometrical roles: the geometry of the photonic architecture defines the optical-state landscape, whereas the illumination–observation geometry determines which of these states become experimentally accessible. Here, the optical-state landscape refers collectively to the spatially, angularly, and spectrally distinct optical response channels supported by the curved system.

## 2. Materials and Methods

### 2.1. Materials

The following materials were used in all experiments: SiO_2_ submicron spheres (diameter 0.264 µm, 5% *w*/*v* aqueous suspension; microParticles GmbH, Berlin, Germany); polystyrene flakes (Sigma-Aldrich, St. Louis, MO, USA; Product No. 331651; melting point 240 °C); commercially available microscope glass slides, planar silver-coated mirrors, glass rods (2 mm in diameter), and polytetrafluoroethylene (PTFE) tape; reagent-grade hydrofluoric acid (HF), ethanol, Rhodamine 6G aqueous solution (10 mg/ml), and distilled water.

### 2.2. Preparation of Planar and Curved Photonic Architectures

#### 2.2.1. Self-Assembly of Planar Silica Opals

Two microscope glass slides were horizontally superposed and separated by a 20 µm thick aluminum-foil spacer. A silica colloidal suspension was deposited using a syringe at the edge of the assembly and drawn into the gap between the slides by capillary action. During evaporation under ambient conditions, colloidal self-assembly occurred within a narrow region adjacent to the slide edge, producing a planar silica opal band approximately 1 mm wide and several centimetres long. After complete evaporation of the water, the slides were separated to expose the planar opal surface for optical investigation.

#### 2.2.2. Self-Assembly of Hemispherical Silica Opals

A SiO_2_ colloidal droplet was deposited onto the tip of a vertically suspended glass rod (2 mm in diameter). The droplet volume was gradually increased by successive additions of the colloidal suspension until the largest stable droplet was obtained immediately before spontaneous gravitational detachment. As previously demonstrated, the diameter of the resulting hemispherical colloidal crystal is governed by the diameter of the supporting glass rod, which determines the maximum stable droplet volume [25].

The droplets were dried under ambient laboratory conditions for 60–120 min, yielding iridescent hemispherical colloidal crystals. Considering the macroscopic thickness of the crystals relative to the 264 nm particle diameter, the structures are estimated to contain several hundred close-packed nanoparticle layers, with the local number of layers varying across the curved geometry. The crystals were readily detached from the supporting glass rod by gently inserting a sharp blade at the crystal–glass interface. Thereafter, all crystal handling and positioning throughout the experiments were performed using the corner of the same blade.

#### 2.2.3. Fabrication of Supported Graded Photonic Heterostructures

Small fragments of polystyrene (typically a few hundred micrograms to a few milligrams) were placed onto a planar silver-coated mirror and heated above the polymer melting point. The molten polymer spread spontaneously over the mirror surface, producing nearly circular polymer films whose diameter, determined by the amount of polymer used, typically ranged from approximately 2 to 7 mm. The hemispherical silica opal was subsequently placed onto the molten polymer film with its convex side facing upward. The assembly was reheated at 250 °C for 30 min, allowing partial polymer infiltration by the previously reported rise infiltration mechanism [26].

During this process, the circular polymer film simultaneously served as the polymer reservoir for infiltration and as the mechanical support of the resulting heterostructure. The procedure yielded supported graded photonic heterostructures without the formation of a continuous polymer overlayer (polymer skin) on the external crystal surface [26].

### 2.3. Optical Interrogation Geometries

#### 2.3.1. Curved Opal–Water Cavities on PTFE Substrates

A planar PTFE substrate was prepared by wrapping PTFE tape around a microscope glass slide. A water droplet approximately 1 mm in diameter was deposited onto the horizontal PTFE surface using a microsyringe. The hemispherical silica opal was positioned adjacent to the droplet with its convex side facing upward and gently pushed until partially immersed. The initial droplet was intentionally kept smaller than the crystal to preserve its orientation during insertion. Additional water was then added using the microsyringe until the crystal became completely immersed, yielding a curved opal–water optical cavity.

#### 2.3.2. Curved Opal–Water Cavities on Mirror Substrates

A water droplet approximately 1 mm in diameter was deposited onto the surface of a horizontally positioned planar silver-coated mirror using a microsyringe. The hemispherical silica opal was placed adjacent to the droplet with its convex side facing upward and was gently pushed until partially immersed. Additional water was subsequently added using the microsyringe until the crystal became completely immersed, yielding a curved opal–water optical cavity supported by the planar mirror.

#### 2.3.3. Conventional Optical Configurations in Air and Bulk Water

Planar and hemispherical silica opals were investigated both in air and after immersion in distilled water. For measurements in air, the planar opals were positioned with their exposed photonic surface facing the fibre-optic probe, whereas the hemispherical opals were positioned with their convex surface facing upward. For measurements in bulk water, the samples were completely immersed in distilled water while preserving the same orientation relative to the probe. Unless otherwise specified, illumination and collection were performed in the conventional retroreflection geometry, with the fibre-optic probe oriented normal to the investigated sample region.

#### 2.3.4. Working-Distance-Dependent Spectral Measurements

Working-distance-dependent UV–Vis measurements were performed by progressively varying the distance between the distal end of the fibre-optic probe and the uppermost point of the investigated photonic surface. For each working-distance-dependent series, all spectra were acquired from the same photonic object, whose structure and orientation relative to the optical axis were maintained unchanged; only the probe–object distance was varied. Consequently, the crystal thickness, defect population, and local structural organization remained invariant throughout each measurement series. Unless otherwise specified, spectra were acquired at working distances ranging from 1 to 7 mm, using increments of 1 mm. For clarity, only selected spectra from these series are displayed in the corresponding figures. For the quantitative analysis of the broad spectral feature near 460 nm in the graded curved photonic heterostructure, the investigated range was extended from 0 to 9 mm in increments of 1 mm, with an additional measurement performed at 0.5 mm. The same general procedure was applied to planar and curved opals immersed in bulk water, curved opal–water cavities supported by planar mirrors, and graded curved photonic heterostructures.

#### 2.3.5. Optical Imaging and Spectroscopy Under Natural Illumination:

Spatial optical responses were imaged under natural illumination using either diffuse skylight or direct sunlight, depending on the investigated configuration.

##### Halo States Imaging

Halo-like optical patterns were observed by introducing angularly separated natural illumination while maintaining optical observation through the microscope.

##### Beam-like Optical Pathways

A water droplet was deposited onto a planar silver-coated mirror and illuminated by direct sunlight near its periphery. The illumination and observation directions were arranged in an approximate retroreflection geometry within a vertical plane containing the droplet centre. The incident sunlight intersected the water–air interface near the droplet–mirror contact region on the side opposite the light source, at an angle of approximately 30° relative to the droplet symmetry axis, corresponding to the surface normal at its apex. The resulting directional beam was most readily visualized when only a narrow strip of the projected solar image illuminated the droplet–mirror system. Restricting the illuminated region substantially reduced background glare without noticeably affecting the beam itself.

##### UV–Vis Measurements Under Natural Illumination

For UV–Vis measurements under natural illumination, the AvaLight-DHc source was switched off, and the central collection fibre of the probe was used to record the optical response. Spectra were acquired with the collection axis oriented at θ = 0° and θ = 30° relative to the local surface normal at the opal apex. Direct sunlight and diffuse skylight were distinguished according to whether the solar disc directly illuminated the sample.

### 2.4. Investigations

Macroscopic morphology was assessed using zoom-camera imaging and optical microscopy. In the optical photographs, the bright white spots correspond to surface gloss produced by specular reflection at the curved liquid–air interfaces and should not be interpreted as particle aggregates.

UV–Vis retroreflection spectra were recorded using an AvaLight-DHc light source and an AvaSpec-ULS2048CL-EVO spectrometer (Avantes, Apeldoorn, The Netherlands), coupled through a 6-around-1 fibre-optic reflection probe consisting of six illumination fibres surrounding a central collection fibre. Each fibre had a core diameter of 400 µm and a nominal numerical aperture of 0.22. The probe head incorporated manufacturer-supplied focusing optics.

The illuminated spot was approximately 1 mm in diameter at a working distance of 1 mm and increased to approximately 2 mm at a working distance of 7 mm, corresponding to illuminated areas of approximately 0.79 and 3.14 mm^2^, respectively. Thus, the illuminated region varied with working distance, as intended in the distance-dependent experiments.

The probe was held in a fixed vertical support, with its optical axis visually aligned normal to the investigated region at the sample apex. Because illumination was delivered through six fibres and focused by the probe optics, the incident field occupied a finite angular range rather than a single incidence angle. Consequently, any reported incidence geometry refers to the nominal orientation of the probe optical axis relative to the local surface normal. No goniometric angular scan was performed, and the angular alignment uncertainty was not independently calibrated.

Commercial household aluminum foil was used as the reflectance reference.

### 2.5. Reproducibility

The hemispherical colloidal photonic crystals fabricated by the hanging-drop self-assembly method were reproducibly obtained, consistent with our previous report describing this fabrication protocol [27]. SEM examination revealed extended regions of locally uniform close-packed ordering, together with occasional packing defects and boundaries between ordered domains, as commonly observed in evaporation-driven colloidal self-assembly. These local imperfections did not prevent the reproducible formation of the overall hemispherical architecture. Likewise, the graded curved photonic heterostructures obtained by partial polystyrene infiltration of the opal, producing a fully infiltrated basal domain and a non-infiltrated apical domain, were reproducibly fabricated with essentially identical optical appearance and spectroscopic behaviour.

The reproducibility of the geometry-dependent optical phenomena depended primarily on the experimental configuration. Superspectrum formation in curved opal–water cavities deposited on PTFE substrates was reproducible but occurred only within a relatively narrow range of opal-to-droplet diameter ratios. In contrast, replacing the PTFE substrate with a planar mirror substantially relaxed these geometrical constraints, allowing reliable superspectrum formation over a considerably broader range of droplet diameters (typically 3–7 mm for curved opals with diameters of 1.5–2 mm).

The characteristic sigmoidal evolution of the geometry-dependent spectral response as a function of working distance was consistently reproduced for both liquid-cavity and solid-state heterostructure configurations. Likewise, UV–Vis reflectance measurements performed under natural illumination yielded highly reproducible spectral responses under identical illumination–observation geometries.

The reproducibility of the spatial optical phenomena varied according to their geometrical requirements. Beam-like optical pathways were readily reproduced once the required droplet–mirror illumination geometry was established, namely direct solar illumination near the droplet–mirror–air contact region, with the illumination and observation directions arranged in an approximate retroreflection configuration. Halo-like optical patterns exhibited lower reproducibility than the spectral responses, particularly in their detailed spatial morphology. Based on our observations, the most probable controlling variables are the local curvature of the liquid–air interface, the position of the opal relative to the droplet edge, and the relative illumination–observation geometry, including the direct or diffuse character of the natural illumination. Because these variables were not varied independently, their individual contributions and the conditions required for deterministic halo formation remain unresolved.

## 3. Results and Discussion

### 3.1. Geometry-Controlled Optical-State Accessibility in Coupled Curved Photonic Systems

Because no general experimental strategy currently exists for the optical interrogation of curved photonic geometries, we present the experimental configurations in a sequence that reflects neither increasing experimental complexity nor a predetermined narrative, but the progressive emergence of a unified physical picture [1]. Each configuration represents the experimental form taken by a question raised by the preceding observation.

We begin by comparing the simplest pair of photonic architectures: planar and curved silica opals. Because they share essentially the same composition, photonic periodicity, and measurement conditions while differing fundamentally in geometry, their comparison isolates the role of geometry in shaping the experimentally accessible optical response.

**Figure 1:**
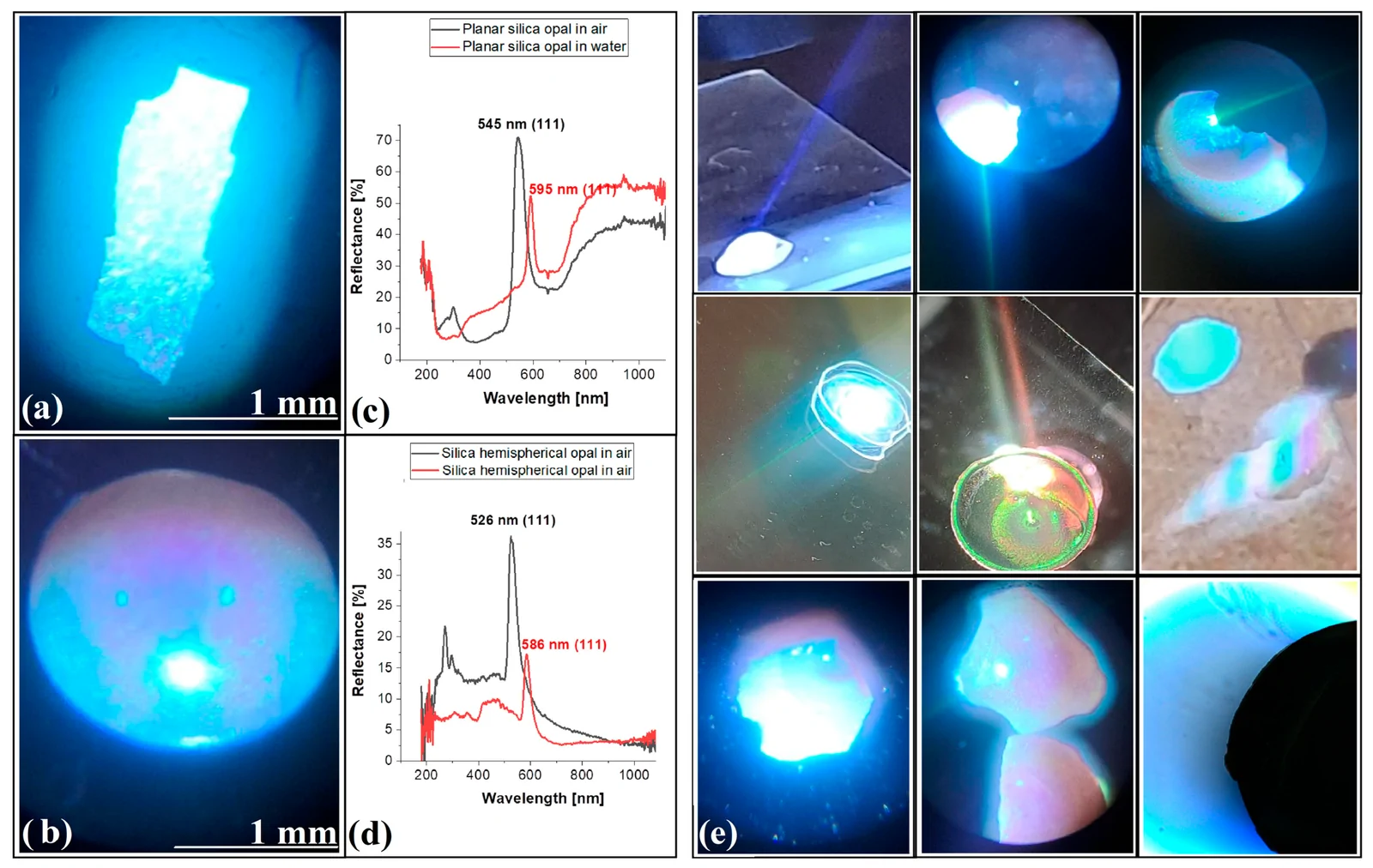
Planar and curved silica opals and their optical responses. (**a**,**b**) Representative optical images; (**c**,**d**) corresponding UV–Vis retroreflection spectra in air and water; (**e**) selected geometry-dependent optical manifestations of curved photonic architectures.

The most striking difference between planar and curved photonic crystals is purely visual. A high-quality planar opal exhibits a single structural colour observed under a unique illumination–observation geometry (Figure 1a). In contrast, a curved photonic crystal displays a geometry-dependent chromatic mapping, revealing a continuous distribution of structural colours across its surface. This mapping forms concentric circular rings for spherical opals (Figure 1b) and progressively deforms into elliptical patterns as the photonic architecture becomes ellipsoidal.

At first sight, this striking visual difference appears to arise naturally from the fundamentally different geometries of the two photonic architectures. A planar photonic crystal possesses a unique surface normal, so that, according to the Bragg condition, a fixed illumination–observation geometry selects a single diffraction wavelength (Figure 1a). In contrast, the continuously varying surface normal of a curved photonic crystal causes different surface regions to satisfy the Bragg condition at different local incidence angles, producing a geometry-dependent chromatic mapping under the same external illumination–observation geometry (Figure 1b).

Surprisingly, this striking visual contrast is largely absent in conventional UV–Vis retroreflection spectroscopy. Despite their fundamentally different geometries and chromatic mappings, planar and curved silica opals exhibit remarkably similar spectral responses under identical illumination–observation conditions (Figure 1c,d). In both air and water, the spectra are dominated by the fundamental (111) Bragg reflection.

Additional experimental observations further deepen this apparent contradiction. Several remarkable optical phenomena—including the previously reported blue-beam effect and ghost images in curved inverse opals [13], analogous green-beam states in curved silica opals, double-beam configurations, beam–halo coupling states, and extended luminous halos—are observed exclusively in curved photonic architectures under specific experimental geometries (Figure 1e).

These observations suggest that conventional spectroscopy samples only a restricted subset of the optical behaviour supported by curved photonic architectures. The coexistence of pronounced visual manifestations—often interpreted as mere experimental artefacts—with weak or absent spectral counterparts shows that visual observation and spectroscopic detection are not necessarily equivalent in curved photonic systems. This mismatch provided the starting point for the experiments that followed.

**Figure 2:**
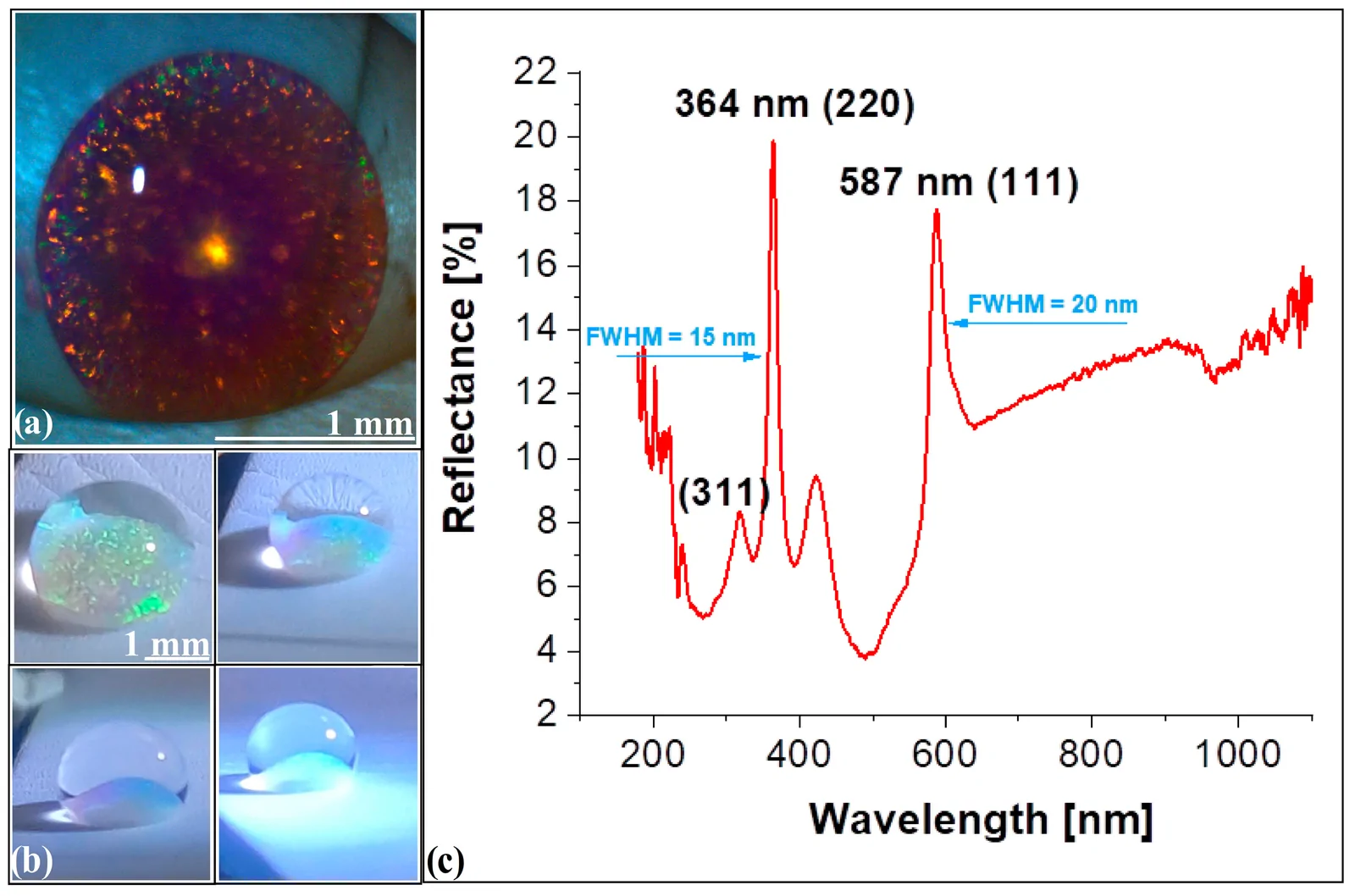
Curved silica opal within a spherical water droplet on a PTFE substrate. (**a**) Optical microscopy image; (**b**) representative photographs; (**c**) UV–Vis superspectrum resolving multiple Bragg reflection bands.

We reasoned that one promising way to explore this mismatch would be to couple the opal’s curved geometry to a second, appropriately chosen optical geometry, by analogy with compound lenses, whispering-gallery resonators, or Fresnel-reflection cavities. To test this possibility, we investigated the simplest such system: a hemispherical silica opal confined within a spherical water droplet deposited on a flat Teflon substrate (Figure 2a,b).

Under these conditions, the conventional single-band response characteristic of the curved opal (Figure 1d) evolves into a highly organized superspectral state (Figure 2c), where multiple narrow reflection bands emerge near 587 nm, 422 nm, 364 nm, and 318 nm. Assuming an average silica sphere diameter of 264 nm (±5%), a refractive index *n* = 1.42 provided by the manufacturer, and an ideal fcc filling factor of 0.74, the theoretically expected reflection bands for a water-infiltrated planar opal are located near 603 nm for the (111) planes, 522 nm for the (200) planes, 369 nm for the (220) planes, 315 nm for the (311) planes, and 301 nm for the (222) planes. The experimentally observed bands at 587 nm, 364 nm, and 318 nm therefore exhibit very good agreement with the theoretically expected (111), (220), and (311) Bragg reflections, respectively.

The close agreement between the measured band positions and the Bragg reflections calculated for a planar water-infiltrated opal raises an immediate question: why does a curved photonic crystal coupled to a liquid optical cavity retain the spectral positions predicted for a planar lattice? The answer appears to lie in the separation between local photonic order and global geometry. Curvature redistributes the orientations of locally ordered crystal domains without substantially altering their lattice spacing, while the liquid cavity modifies how their diffraction channels are coupled and simultaneously expressed, with selected contributions appearing amplified in the measured spectrum. The spectral positions therefore remain anchored to the local Bragg conditions, whereas their relative intensities, linewidths, and simultaneous accessibility are governed by the global coupled geometry.

Most striking is the unusually intense (220) reflection, whose intensity surpasses that of the fundamental (111) reflection despite representing a higher-order Bragg reflection [28]. Equally remarkable are the exceptionally narrow spectral widths of the resolved bands, with full widths at half maximum of approximately 20 nm for the (111) reflection and 15 nm for the (220) reflection. Such narrow linewidths are difficult to explain solely by the reduced refractive-index contrast between silica and water or by an exceptionally high degree of colloidal ordering, since hanging-drop opals, although containing crystalline monodomains extending over tens of micrometres, nevertheless retain an overall polycrystalline organization [25].

The asymmetric sawtooth-like profile of the dominant (111) reflection, the apparent absence of the expected (200) and (222) contributions from the measured spectrum despite the excellent agreement observed for the remaining principal fcc reflections, and the intense reflection band near 422 nm, which cannot be assigned to the principal Bragg reflections of an ideal water-infiltrated fcc opal, further suggest that optical coupling within the photonic liquid cavity selectively weights and spectrally reshapes the contributions entering the detected signal.

Operationally, the cavity acts as an optical preprocessor between the photonic structure and the detection system: it transforms part of the spatially and angularly distributed optical response into a wavelength-dependent signal accessible within a single fibre-coupled measurement. The superspectrum should consequently not be regarded simply as an intrinsic spectrum of the immersed opal, but as the measurable output of the coupled opal–droplet–illumination–collection system. Seen from this perspective, even the apparently intrinsic spectrum of a planar opal represents the response of a coupled opal–illumination–collection system. In this case, however, no additional cavity coupling is required because conventional UV–Vis interrogation is already geometrically matched to the planar architecture.

**Figure 3:**
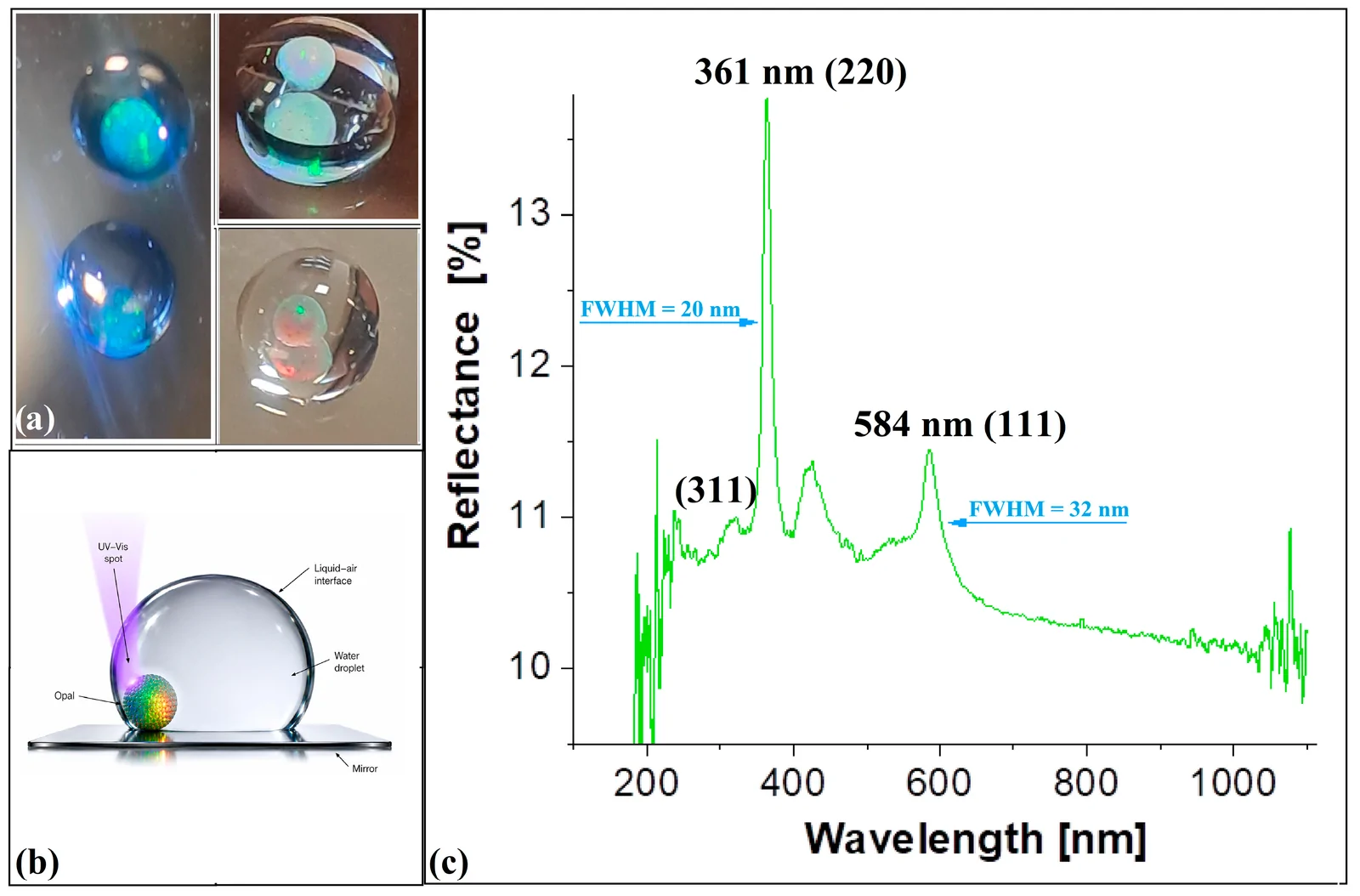
Curved silica opal–water cavity on a planar mirror. (**a**) Representative configurations; (**b**) schematic experimental geometry; (**c**) UV–Vis superspectrum obtained under favourable coupling conditions.

If coupling a curved photonic crystal to a simple liquid cavity reveals optical responses inaccessible to conventional spectroscopy, a more complex coupled cavity might be expected to reveal an even richer optical behaviour. Motivated by this possibility, the Teflon substrate was replaced by a planar silver-coated mirror (Figure 3a).

Surprisingly, the superspectral state is readily reproduced in this more complex cavity configuration (Figure 3c), exhibiting only minor spectral changes relative to the original water-droplet-on-Teflon system. Moreover, the conditions for its observation become less restrictive with respect to droplet size. Instead, the critical requirement becomes geometrical: retroreflection must occur at the opal–water interface. This condition is satisfied either when the opal is positioned near the droplet edge in larger droplets or when it is centred within droplets having a diameter comparable to that of the opal (Figure 3b).

Reversing the orientation of the curved opal relative to the mirror or disrupting its structural integrity suppressed the fully developed superspectral state, restoring a more conventional water-infiltrated opal response. Nevertheless, the principal Bragg reflections remained simultaneously observable, although only partially resolved. These intermediate responses show that superspectrum formation is not an all-or-nothing event: the curved architecture preserves multiple diffraction channels, while their relative prominence in the measured spectrum and degree of spectral resolution vary with the quality of geometrical coupling.

**Figure 4:**
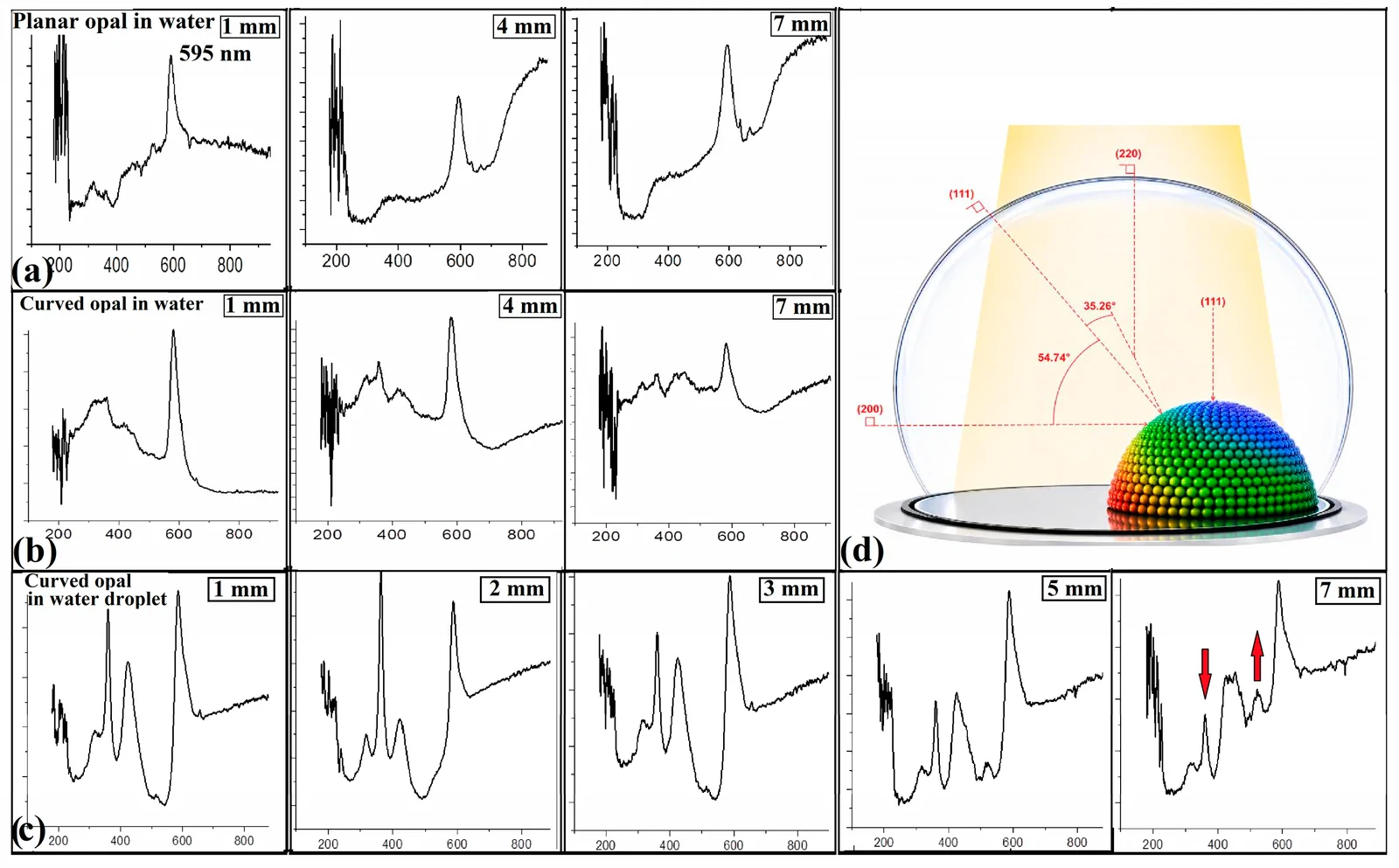
Working-distance-dependent UV–Vis spectra of (**a**) a planar opal in water, (**b**) a curved opal in water, and (**c**) a curved opal within a water droplet on a planar mirror; (**d**) proposed geometrical relation between the relevant crystallographic directions.

We next investigate whether the experimentally observed optical response can be continuously tuned through the collection geometry. As previously demonstrated [29], varying the working distance continuously changes the optical volume sampled by the fibre-optic probe and, consequently, its acceptance cone, field of view, and angular sampling of the curved photonic surface. Within the present framework, the working distance therefore provides a convenient experimental parameter for continuously selecting which simultaneously exposed optical states contribute to the measured spectrum. The working distance between the opal surface and the fibre-optic probe was therefore systematically varied for two conventional configurations—planar and curved opals immersed in bulk water—and for the coupled photonic liquid-cavity configuration formed by a curved opal confined within a water droplet on a planar mirror. Under conventional measurement conditions, both the planar (Figure 4a) and the curved (Figure 4b) opals exhibit essentially the same behaviour.

Varying the working distance produces only minor spectral changes, while the (111) Bragg reflection remains nearly unchanged, indicating that the collection geometry alone has little influence on the conventional optical response. The only notable exception is a broad spectral contribution extending across the 400–500 nm region, observed exclusively for the curved opal. This feature is already present in air (Figure 1d) and persists after water infiltration, exhibiting essentially no spectral shift despite the substantial change in effective refractive index. This broad contribution therefore appears to originate from an optical mechanism other than Bragg diffraction.

A fundamentally different behaviour emerges for the coupled photonic liquid cavity. As the working distance is progressively increased, an additional narrow reflection band develops near 520 nm (Figure 4c), close to the theoretically expected position of the (200) Bragg reflection for a water-infiltrated fcc opal. Simultaneously, the intensity of the (220) reflection decreases monotonically, producing a pronounced anticorrelation between the two bands (red arrows).

This continuous spectral evolution indicates a progressive redistribution of optical intensity between two simultaneously accessible optical states rather than the appearance of a new diffraction process. Here, redistribution refers to the changing relative coupling of the (200) and (220) contributions into the fibre-collected signal, without implying direct energy transfer between the two diffraction channels. One possible geometrical interpretation (Figure 4d) involves simultaneous direct and mirror-reflected illumination of the same crystal region, where the geometrically complementary (220) and (200) plane families (35.3° and 54.7° with respect to the (111) direction) may contribute differently to the collected response through distinct recirculated optical pathways.

Such behaviour appears to require the combined action of a reflective substrate, an extended illumination source, and a curved air–liquid interface supporting multiple total internal reflections, which together promote optical recirculation within the coupled cavity.

Changing the working distance continuously modifies which of these recirculated optical pathways are coupled into the fibre acceptance cone, thereby continuously reshaping the measured spectrum. The superspectrum may therefore represent the limiting case of the same geometrical evolution, corresponding to the maximum measured contribution of the (220) band, while the contribution near 520 nm, assigned to the (200) reflection, is not resolved in the fibre-collected spectrum.

The experiments discussed so far describe the optical-state landscape through fibre-coupled spectroscopy, where the collected optical field is integrated into a single wavelength-dependent signal. This representation, however, contains little information about the spatial organization of the simultaneously accessible optical states. We therefore next examine the same coupled photonic system by direct optical imaging, allowing the geometrical distribution of the emerging optical field to be observed directly.

**Figure 5:**
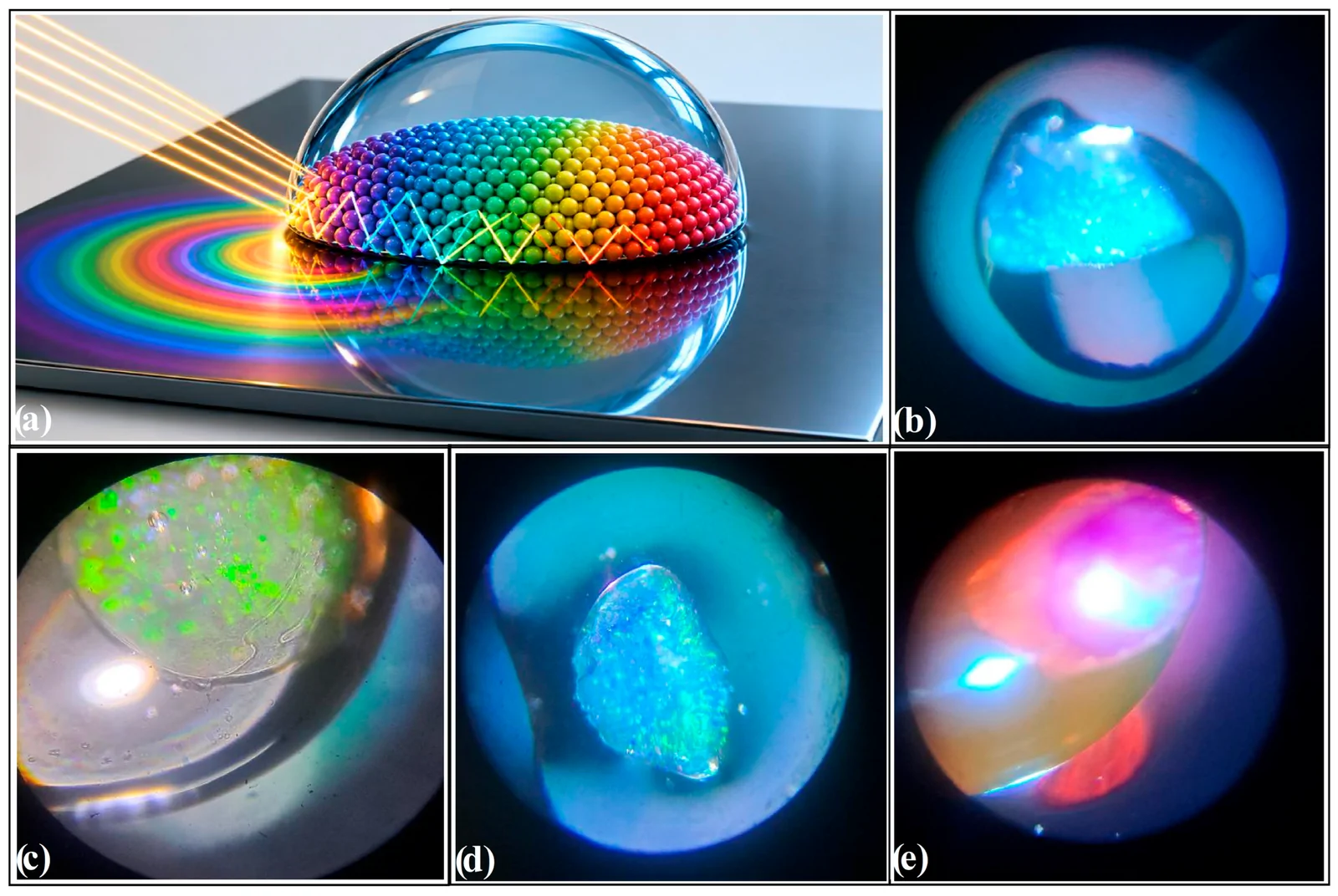
Halo states under oblique illumination–observation geometry. (**a**) Schematic representation of possible optical pathways; (**b**–**d**) curved opals in water; (**e**) curved opal in Rhodamine 6G solution.

In conventional retroreflection microscopy, where the illumination and observation channels are spatially superposed (Figure 1a,b,e and Figure 2a), the coupled photonic system appears primarily as localized coloured regions. The addition of angularly separated natural sunlight revealed a qualitatively different response: well-defined halo-like optical structures surrounding the curved photonic crystal (Figure 5a–d).

Substituting water with Rhodamine 6G within the cavity (Figure 5e) does not suppress halo formation, demonstrating that the phenomenon is not specific to pure water and remains robust to substantial changes in the cavity medium.

Remarkably, the most highly organized halo states occur under essentially the same geometrical conditions that favour superspectrum formation. In both cases, the most prominent observable response is associated with the region between the curved opal surface and the droplet edge, close to the liquid–air interface. This spatial correspondence suggests that the halo-like patterns and the superspectrum may represent complementary spatial and spectral manifestations of geometry-dependent optical-state selection within the coupled cavity. Nevertheless, the present experiments establish neither a one-to-one correspondence between the two phenomena nor a deterministic mechanism of halo formation.

**Figure 6:**
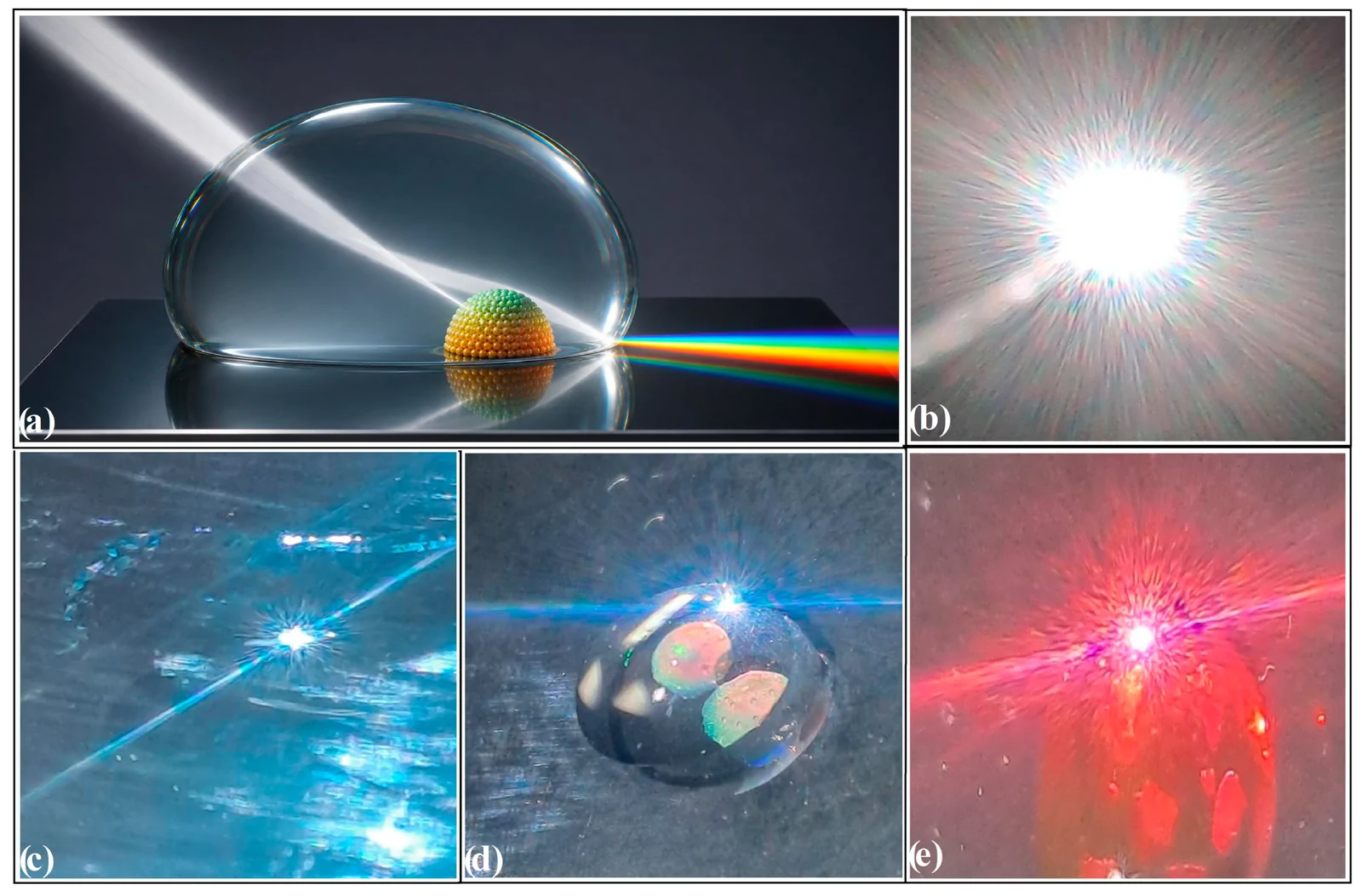
Geometry-selected quasi-collimated beam at the cavity periphery. (**a**) Proposed droplet-mediated beam-redirection geometry; (**b**) conventional solar reflection; (**c**) water droplet; (**d**) droplet containing a curved opal; (**e**) Rhodamine 6G droplet.

The optical response changes qualitatively, however, when diffuse natural illumination is replaced by direct sunlight. The halo states described above were observed under diffuse illumination, which provides a broad distribution of incident directions and permits multiple optical pathways through the coupled cavity. Under direct sunlight, by contrast, both illumination and observation were confined to the cavity periphery (Figure 6a), where a distinct optical phenomenon emerged.

Under these conditions, a water droplet deposited on a planar mirror redirects part of the incident sunlight into a quasi-collimated beam propagating parallel to the mirror surface (Figure 6a,c), whereas the mirror alone produces only conventional specular reflection and an aperture-diffraction starburst (Figure 6b).

As previously suggested [13], the droplet may act as a pseudo-cylindrical lens that redirects part of the incident light into a highly directional optical pathway. Introducing a curved opal into the droplet (Figure 6d) produces no obvious change in the beam morphology, although the limited sensitivity of the present imaging configuration may mask subtle effects.

Replacing water with Rhodamine 6G, however, causes the beam to acquire the characteristic fluorescence colour of the dye (Figure 6e), demonstrating that the propagating optical state carries information about the optical medium through which it travels. Together with the halo states, these observations show that the coupled cavity geometry supports multiple geometry-selected propagation regimes, including cavity-confined recirculating fields and highly directional peripheral optical pathways. Although no detectable modification of the beam by the curved opal was observed, this configuration establishes a potentially useful directional interrogation geometry for materials introduced into the cavity.

**Figure 7:**
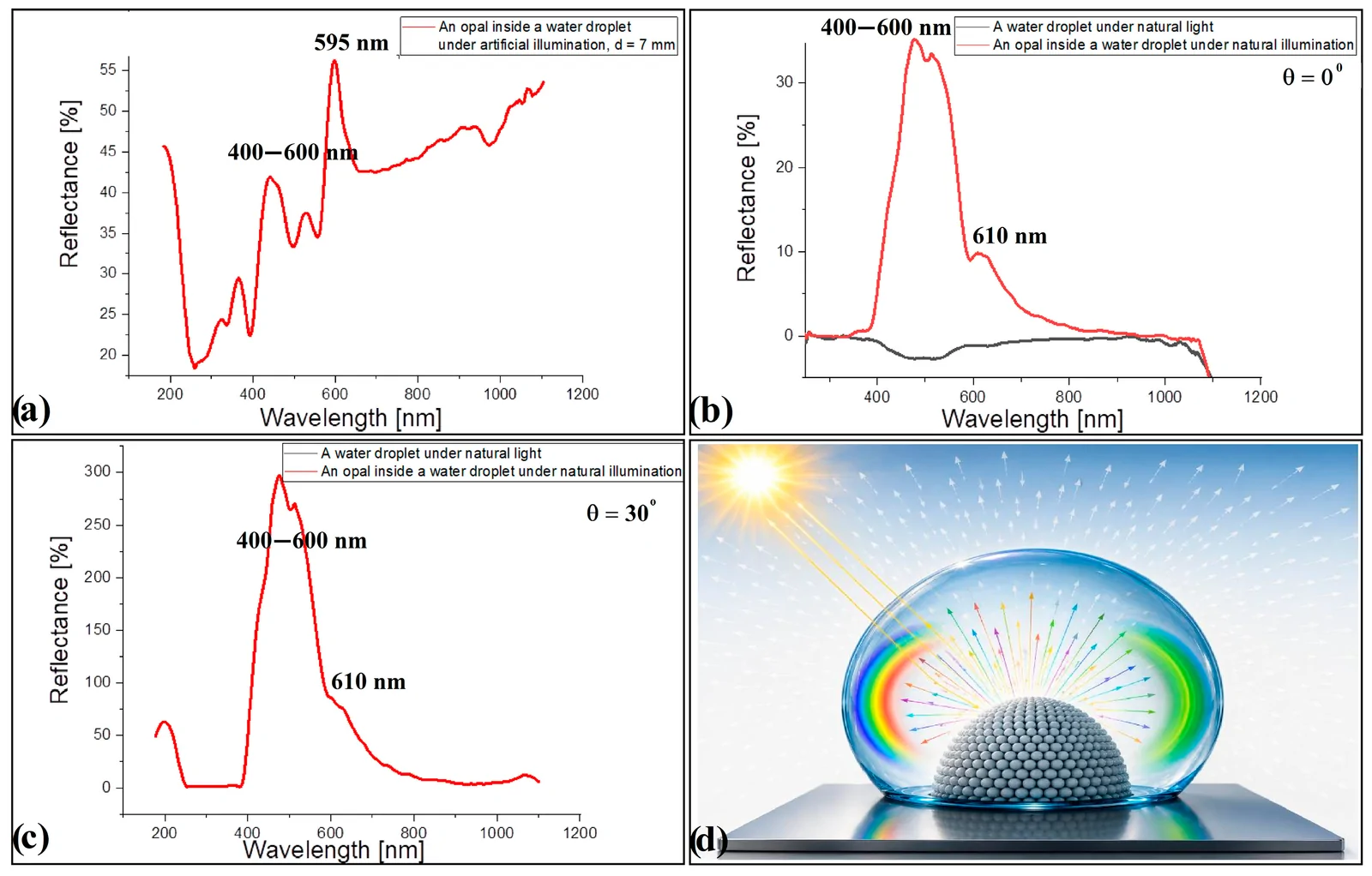
Optical response under laboratory and natural illumination. (**a**) Reference spectrum under artificial illumination; (**b**,**c**) spectra under natural illumination collected at θ = 0° and 30°, respectively; (**d**) conceptual representation of direct and diffuse natural illumination.

The halo states (Figure 5) and the beam-like optical states (Figure 6) are both generated under natural illumination, the former being excited predominantly by diffuse skylight and the latter by direct solar illumination. Although these observations reveal distinct geometry-selected optical states, they provide little information about their corresponding spectral signatures because the Sun itself serves as the illumination source. This naturally raises the question of whether natural illumination also modifies the spectroscopically accessible optical states. To address this question, UV–Vis measurements were performed under natural solar illumination using controlled observation geometries (Figure 7).

Replacing the conventional laboratory light source (Figure 7a) with natural illumination (Figure 7b) substantially alters the measured optical response. Under diffuse skylight (Figure 7b), the spectrum is dominated by a broad structured band extending throughout the 400–600 nm region, whereas the relative contribution of the (111) Bragg reflection is markedly reduced compared with conventional laboratory measurements (Figure 7a). Changing only the observation geometry from normal collection (θ = 0°) to oblique collection (θ = 30°) produces a further redistribution of the reflected optical intensity (Figure 7b,c). Under oblique observation, the (111) reflection becomes barely distinguishable, while the total reflected intensity increases by nearly one order of magnitude. These changes occur without modifying the photonic architecture itself, demonstrating that the illumination–observation geometry alone can profoundly reshape the experimentally accessible optical response.

These observations extend the geometrical framework established above. Throughout the preceding experiments, changing the cavity geometry or the collection geometry continuously redistributed the experimentally accessible optical states. Natural illumination introduces an additional level of geometrical complexity by combining continuously varying contributions from direct solar radiation and diffuse skylight within a three-dimensional illumination environment. Consequently, spectral equivalence between laboratory and natural illumination should not be interpreted as optical equivalence. Rather, natural illumination selectively excites and reveals different subsets of the optical-state landscape according to the relative contributions of direct sunlight, diffuse skylight, and the observation geometry.

This conclusion has direct implications for biological photonic systems. Unlike laboratory measurements performed under fixed illumination conditions, natural photonic architectures operate continuously within a dynamically evolving optical environment. If optical-state accessibility is indeed governed by geometry, as the present results consistently indicate, natural illumination becomes an active component of photonic function rather than merely its excitation source. Investigating curved photonic architectures under natural illumination therefore extends their characterization beyond intrinsic material properties toward the study of their functional optical behaviour in realistic biological environments.

Remarkably, the experimental geometries favouring superspectrum formation (Figure 3b), halo generation (Figure 5a), beam-like propagation (Figure 6a), and the modified spectral responses observed under natural illumination (Figure 7) all share a common characteristic: the optical response depends critically on the geometrical relationship between the curved photonic architecture, its surrounding optical environment, and the illumination–observation geometry. Taken together, these experiments demonstrate that curved photonic architectures do not possess a unique optical response. Instead, they support a geometrically generated landscape of optical states whose experimental manifestation depends on the coupled geometry of the photonic architecture, the surrounding optical environment, and the illumination–observation configuration.

### 3.2. Geometry-Guided Engineering of Optical Functionality in Coupled Curved Photonic Systems

**Figure 8:**
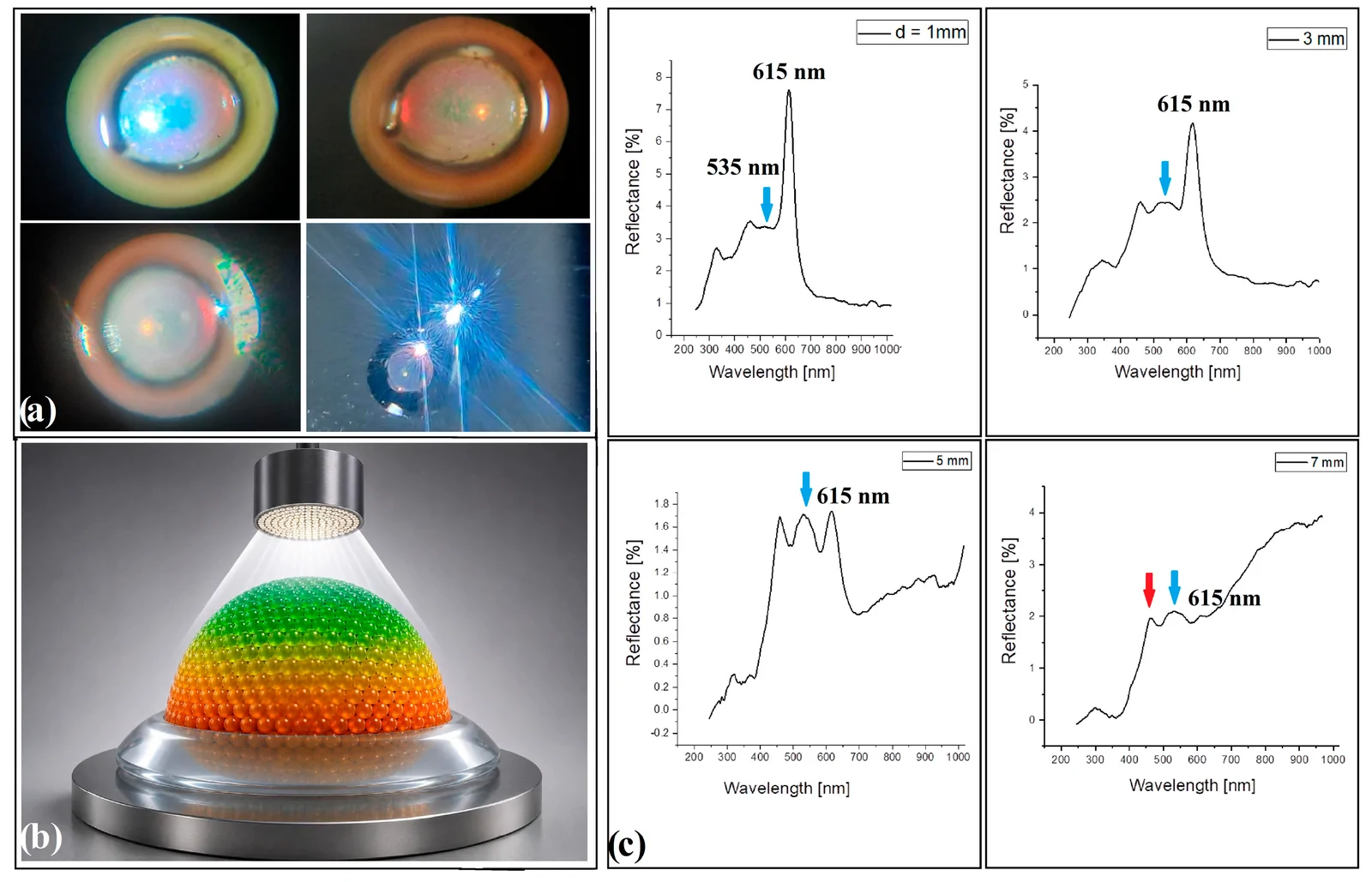
Graded curved photonic heterostructure. (**a**) Optical appearance under different illumination geometries; (**b**) schematic fibre-coupling geometry; (**c**) UV–Vis spectra recorded at different working distances.

The geometry-dependent optical phenomena described above naturally raise a practical question: can the same geometrical principle be implemented in a structurally stable solid photonic architecture? To address this question, we developed a graded curved photonic heterostructure (see Section 2.2.3) consisting of a fully polystyrene-infiltrated silica opal at the base and the original non-infiltrated silica opal at the apex, thereby preserving the curved photonic geometry while replacing the liquid cavity with a permanent solid-state architecture. Figure 8a illustrates the optical appearance of the heterostructure under four representative illumination geometries: coaxial microscope illumination, diffuse ambient illumination, and the solar illumination geometries employed previously in Figure 5a and Figure 6a.

Several optical signatures characteristic of the liquid cavity reappear, including circular spectral mapping, halo-like emission along the outer polymer ring, and, most remarkably, two intersecting beam-like emissions together with their reflections in the underlying mirror. Their reappearance demonstrates that the geometry-dependent optical functionality is largely preserved following transfer to the solid-state architecture, although the emergence of two beams instead of one suggests an additional optical process, possibly involving interference between the polymer layer and the underlying mirror.

From a spectral perspective, the response of the graded heterostructure is governed by the coexistence of two photonic domains with different effective refractive indices and by the geometry-dependent coupling of their optical fields into the fibre-optic probe. Varying the probe distance continuously changes the relative contribution of these two domains to the collected signal, thereby selecting different subsets of optical pathways within the same illumination–observation geometry (Figure 8b). This behaviour is clearly evidenced by the UV–Vis spectra shown in Figure 8c. As the probe distance is increased from 1 to 7 mm, the (111) Bragg reflection of the non-infiltrated curved opal, centred near 535 nm, progressively increases, whereas the (111) reflection of the polystyrene-infiltrated domain, centred near 615 nm, gradually decreases (blue arrow).

**Figure 9:**
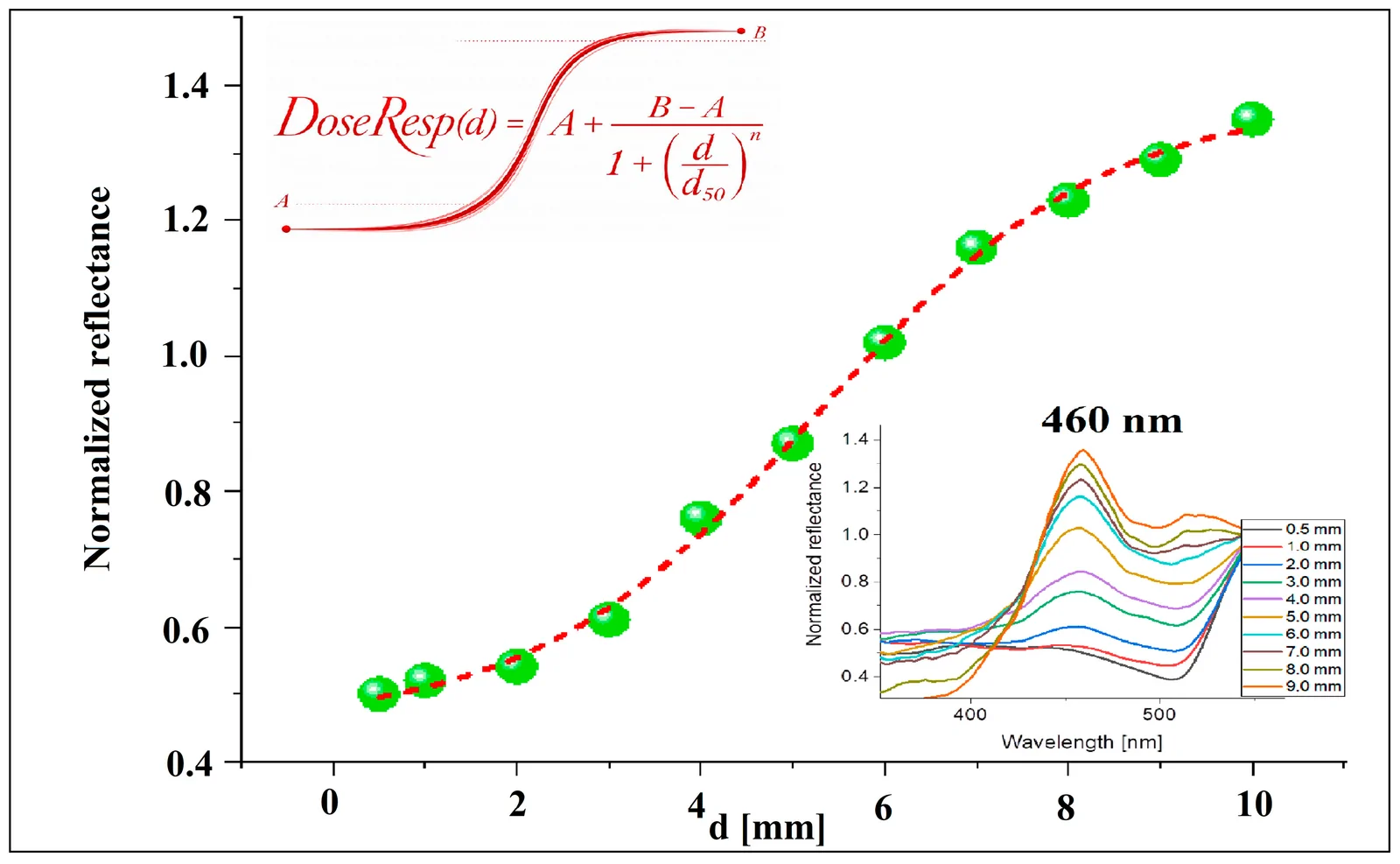
Normalized intensity of the broad spectral feature near 460 nm as a function of working distance, with the sigmoidal fit and the corresponding normalized spectra shown in the insets.

We also recover the broad spectral feature previously observed for the curved crystal in air, in bulk water, and within a water droplet, extending across the 400–500 nm region and centred near 460 nm (red arrow). Its persistence under different conditions, its absence from planar crystals, and the absence of a measurable spectral shift identify it as a persistent experimental feature associated with the curved architecture, but do not establish its microscopic origin. In particular, the present measurements do not distinguish conclusively among incoherent or multiple scattering, whispering-gallery-type or other low-Q cavity modes, and geometry-dependent phase-matching effects. Its reflectance normalized to the (111) band nevertheless varies monotonically with working distance and is empirically described by a sigmoidal DoseResp function (Figure 9).

This behaviour suggests a gradual transition between two limiting collection regimes, with the inflexion point potentially marking the distance at which the fibre acceptance cone receives comparable optical contributions from the non-infiltrated and polystyrene-infiltrated photonic domains. Although no specific application is immediately evident, the heterostructure simultaneously exhibits two reproducible working-distance-dependent responses: the anticorrelated redistribution between the (111) bands of the non-infiltrated and polystyrene-infiltrated domains, and the monotonic evolution of the broad spectral contribution near 460 nm. Their coexistence provides multiple spectral degrees of freedom that may be useful for the development of geometry-responsive optical devices.

## 4. Outlook

The present findings suggest that the experimentally observable response of a photonic structure is determined not by the structure alone, but by the geometry through which the structure, its surrounding medium, illumination, and observation are coupled. A given configuration therefore provides access to only a particular region of the optical-state landscape, whereas a different coupling geometry may render another set of states simultaneously observable. Although demonstrated here using curved photonic crystals, this principle may extend to other wave-based systems in which spatially distributed states must be coupled to a restricted detection geometry, including plasmonic and metamaterial architectures, acoustic systems, and possibly quantum wave phenomena.

From this broader perspective, curvature should not necessarily be regarded as a unique physical mechanism. Rather, it is an exceptionally efficient generator of phenomenological diversity. By continuously varying local orientations, interfacial relationships, and propagation directions within a single object, curvature creates multiple opportunities for coupling, redirecting, and selectively observing optical states. Curved photonic systems thus provide an experimentally accessible and visually striking platform in which the consequences of coupled geometry become particularly evident.

The inverse, infiltrated, and compositionally graded curved opals developed in this work may also be regarded as elementary units for constructing photonic architectures of substantially greater complexity. Convex, concave, and planar photonic elements could be combined and organized in three dimensions so that their individual optical-state landscapes become mutually coupled rather than merely superposed. The resulting response would then belong neither to any single constituent nor to their simple geometrical assembly, but to the complete photonic network together with the medium, illumination, and observation geometry through which it is interrogated.

**Figure 10:**
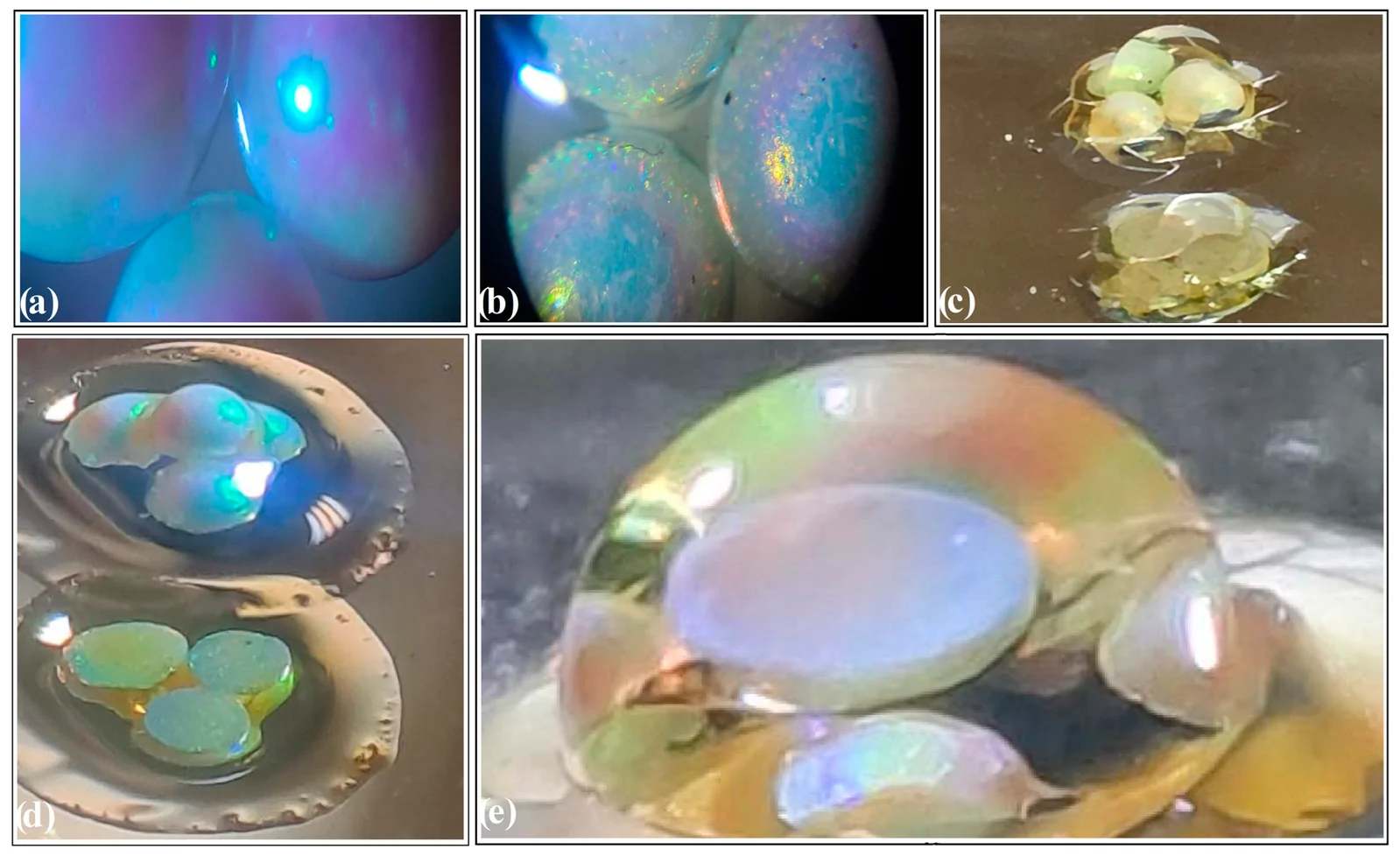
Engineering of complex curved photonic architectures. (**a**) Three mutually tangent convex silica opals; (**b**) corresponding assembly of graded infiltrated opals; (**c**) water or colloidal droplet stabilized within the central cavity; (**d**) extension of the assembly along the third dimension; (**e**) concave–convex photonic units coupled within a common HF droplet cavity.

A simple progression toward such networks is illustrated in Figure 10. Three mutually tangent convex opals (Figure 10a) can be chemically transformed into an ordered assembly of graded infiltrated opals while preserving their collective organization (Figure 10b). Their central interstitial cavity naturally stabilizes a spherical water droplet (Figure 10c), thereby introducing an additional curved interface and a shared optical coupling medium. Alternatively, a colloidal droplet placed within this cavity could self-assemble into a new photonic structure whose size and morphology are constrained by the surrounding units. The geometrical arrangement can be extended into the third dimension (Figure 10d), producing networks of communicating interstitial cavities and curved photonic interfaces. Further diversity may be introduced by coupling convex and concave photonic units through a common HF droplet (Figure 10e). Such a configuration could simultaneously establish multiple light-propagation and reflection pathways, optically couple structures having opposing curvatures, and chemically transform selected constituents of the assembly. These examples suggest a possible transition from the synthesis of individual curved photonic objects toward the design and optical engineering of reconfigurable three-dimensional photonic networks.

In one important respect, such networks may resemble biological photonic systems more closely than conventional engineered materials. Numerous organisms employ curved, hierarchical, and environmentally coupled photonic architectures to produce angle-dependent structural colours and complex visual signals. Their optical function cannot always be attributed to morphology alone, because the observed signal also depends on how the biological architecture interacts with its surrounding medium and with the illumination–observation environment. Geometry is therefore not merely the shape of the photonic structure; it participates in converting an underlying distribution of optical states into a functionally observable signal.

By contrast, biomimetic materials engineering has often concentrated primarily on reproducing the structure, composition, and morphology responsible for biological coloration. Even a highly faithful structural replica, however, may not reproduce the complete optical function if the geometrical coupling among the structure, its external medium, illumination, and observer is omitted. Biomimicry of complex optical functionality may consequently require the reproduction not only of a material architecture, but also of the relational conditions under which that architecture operates. In this context, the graded heterostructure demonstrated here represents an elementary artificial model for investigating how curved geometry and material composition jointly transform observation conditions into measurable optical information. The three-dimensional assemblies proposed in Figure 10 would extend this principle from an individual heterostructure to networks in which optical function emerges collectively from interacting photonic units and shared external media.

This relational character also has consequences for how such systems are explored experimentally. Complex phenomena are not always revealed most effectively by the systematic variation in a single parameter within a fixed configuration [1]. Such an approach can map a given experimentally accessible region with great precision, yet remain insensitive to optical states excluded by the geometry of that configuration. In systems whose observable response changes qualitatively when the coupling geometry is altered, a deliberately broader and less sequential exploration of configurations may therefore be especially fertile. Here, successive experiments involving different curved structures, external media, substrates, working distances, and illumination–collection arrangements exposed distinct manifestations that could not initially be reduced to incremental variations in a single measurement.

Their connection became apparent only when these configurations were recognized as different geometrical probes of the same underlying optical-state landscape. What may appear methodologically nonsystematic at the level of individual experimental arrangements can therefore reveal a deeper order at the level of the coupled system. The challenge is not to replace systematic investigation with unconstrained experimentation, but to determine when the experimental geometry itself must become one of the objects being explored. For curved and increasingly complex photonic architectures, this shift may be essential both for discovering their optical functionality and for learning how to engineer it.

However, the purpose of this work was never to catalogue every optical effect that arises in photonic cavities, but rather to draw attention to their very existence. There is an entire world of optical universes hidden inside a curved photonic crystal placed within a droplet of water resting on a flat mirror—a world far beyond the present monotony of the planar.

## 5. Conclusions

The results presented in this work show that curved photonic architectures do not possess a unique experimentally observable optical response. Instead, the curved photonic architecture defines a landscape of optical states, while the coupled geometry of illumination, observation, and the surrounding optical environment determines which subsets of this landscape become experimentally accessible. In this sense, the curved liquid cavity performs a measurement-enabling transformation: it redirects and spectrally encodes selected spatially and angularly distributed optical contributions into the limited collection channel of the fibre-optic probe. A single measured spectrum may therefore contain optical states that remain inaccessible when the same curved photonic structure is interrogated without the coupled cavity.

The same principle can be transferred from liquid-confined systems to structurally stable photonic architectures. Graded curved photonic heterostructures preserve key optical signatures of the liquid systems and allow their spectral contributions to be continuously selected through the collection geometry. Curved photonic architectures therefore provide a platform in which geometry becomes an active design parameter for selecting, coupling, and experimentally accessing optical states.

## Data Availability

The original contributions presented in this study are included in the article. Further inquiries can be directed to the corresponding authors.
